# Senescence-Associated Vacuoles, a Specific Lytic Compartment for Degradation of Chloroplast Proteins?

**DOI:** 10.3390/plants3040498

**Published:** 2014-11-11

**Authors:** Cristian A. Carrión, Dana E. Martínez, M. Lorenza Costa, Juan José Guiamet

**Affiliations:** Instituto de Fisiología Vegetal (INFIVE, CONICET-UNLP), cc327, La Plata 1900, Argentina; E-Mails: ccarrion@agro.unlp.edu.ar (C.A.C.); danamartinez@conicet.gov.ar (D.E.M.); lorenzacosta@agro.unlp.edu.ar (M.L.C.)

**Keywords:** senescence-associated vacuoles, leaf senescence, chloroplast degradation, proteases, Rubisco, SAG12

## Abstract

Degradation of chloroplasts and chloroplast components is a distinctive feature of leaf senescence. In spite of its importance in the nutrient economy of plants, knowledge about the mechanism(s) involved in the breakdown of chloroplast proteins is incomplete. A novel class of vacuoles, “senescence-associated vacuoles” (SAVs), characterized by intense proteolytic activity appear during senescence in chloroplast-containing cells of leaves. Since SAVs contain some chloroplast proteins, they are candidate organelles to participate in chloroplast breakdown. In this review we discuss the characteristics of SAVs, and their possible involvement in the degradation of Rubisco, the most abundant chloroplast protein. Finally, SAVs are compared with other extra-plastidial protein degradation pathways operating in senescing leaves.

## 1. An Overview of Leaf Senescence

Senescence is typically described as a highly organized process characterized by degradation of macromolecules and cellular structures, ultimately leading to cell, organ and/or plant death [1,2]. Typically, the progression of senescence is assessed through parameters related to the loss of chloroplast integrity (e.g., chlorophyll and protein content) and functioning (e.g., photosynthesis, potential quantum yield of photosystem II). This reflects that the breakdown of chloroplasts is an important process during leaf senescence, and integral to the role of senescence in nutrient redistribution [3,4,5]. Leaves constitute an important store of nutrients, particularly nitrogen, which are mostly held in the photosynthetic apparatus [6]. Within leaves, most N resides in chloroplasts [6]. The degradation of photosynthetic proteins during senescence releases large amounts of N in a readily remobilizable form (*i*.*e*., amino acids), therefore senescence represents a salvage mechanism to recycle chloroplast nitrogen to actively growing organs of the plant (e.g., seeds) or to N reserves in the bark of deciduous trees [3,4,5]. In cereals, up to 80% of N harvested in the grains may come from senescence-associated redistribution of leaf protein N [6]. Given the importance of the breakdown of chloroplast proteins in the nitrogen budget of leaves, crops, and, eventually, global nutrient cycles [7], it is not surprising that senescence-associated changes in other organelles or cell compartments have received much less attention [8,9,10].

## 2. Chloroplast Degradation in Senescing Leaf Cells

Although the main focus of this review is on Senescence-Associated Vacuoles, given their presumed role in chloroplast protein degradation, a brief summary of breakdown processes taking place in senescing leaves seems warranted to provide a context of the developmental scenario where these vacuoles occur. During senescence, massive breakdown of chloroplasts results in the almost complete disappearance of recognizable plastids at late stages of senescence [11,12]. Many studies on chloroplast ultrastructure have documented extensive disorganization of the thylakoid membranes, with a decrease in grana stacking and dilation of thylakoids [11,13]. As thylakoids break down, osmiophillic globuli (plastoglobuli) containing products of membrane degradation appear, reaching relatively large numbers in severely deteriorated chloroplasts [11,12,13]. Eventually, chloroplast structure becomes completely disrupted at late stages of senescence [11]. The ultrastructural changes described above are the consequence of wide-range macromolecule degradation processes. Ultrastructural changes in thylakoids parallel the degradation of photosystem proteins [12,13,14], thylakoid galactolipids [12,15] and loss of chlorophylls [12,16,17], which result in impaired photosynthetic electron transport [13,18]. The stromal proteins of plastids, including Rubisco and other components of the C3 photosynthetic pathway [12,15], glutamine sunthetase II [19], and even sulfur assimilating enzymes [20] are also degraded, and their breakdown may start even before changes in the photochemical apparatus (e.g., chlorophyll loss) can be detected. The relative onset and rate of degradation of stromal *vs*. thylakoid proteins and chlorophyll may vary (e.g., [21]), which may help adjust the remaining photosynthetic capacity of a leaf in the course of senescence to environmental conditions (e.g., irradiance). This highlights the possibility that different proteolytic mechanisms are responsible for the fate of these different photosynthetic proteins.

The degradation of the membrane-bound proteins of photosystem II has been well studied in the context of the repair cycle following photoinhibition of PSII [22,23,24,25,26] and acclimation of the light-harvesting antenna to increased irradiance [27,28,29]. There is evidence that chloroplast proteases of the Deg and FtsH families are involved in the degradation of photodamaged D1 protein [22,23,24,25,26], and because photoinhibition persists during senescence [30], this mechanism might account for D1 breakdown in senescing chloroplasts. The light-harvesting proteins of PSII are degraded during acclimation to high irradiance, possibly through the operation of a metalloprotease [27,28]. *In vitro* experiments implicated FtsH6 in the degradation of the light-harvesting Lhcb2 protein [29], however, this could not be substantiated *in vivo* [31].

In many species, stromal protein degradation is apparently responsible for the marked decrease in photosynthetic activity during senescence, but, perhaps more important, Rubisco also constitutes the single most important source of remobilizable nitrogen in leaves of most species. Thus, understanding chloroplast protein and, particularly Rubisco, degradation, holds the promise of extending photosynthetic capacity or providing a handle to manipulate nutrient redistribution. Unfortunately, data linking a particular protease or a proteolytic pathway to the breakdown of stromal chloroplast proteins is often only correlative, or otherwise inconclusive.

In spite of the intense search for within-the-chloroplast degradation of Rubisco and other stromal proteins, there is still no convincing evidence to implicate chloroplast proteases in Rubisco degradation (reviewed in [14,32]). Moreover, recent findings showing Rubisco and other chloroplast proteins in vesicular structures outside the plastid (*i*.*e*., Rubisco-Containing Bodies and Senescence-Associated Vacuoles [33,34,35]) supports the idea that plastidial degradation of Rubisco may be a minor proteolytic pathway for the breakdown of chloroplast stromal proteins in senescing leaves, whereas extra-plastidial pathways may be more important. These extra-plastidial pathways will be discussed in detail below.

## 3. Involvement of the Central Vacuole

Several reports have suggested that protein degradation in senescing leaves might occur within the central vacuole [36,37,38]. This is consistent with the fact that the central vacuole is an acidic compartment with high endopeptidase and carboxipeptidase activity [39,40], and that a number of senescence-associated proteases localize to the central vacuole [41,42,43], supporting its potential involvement in chloroplast protein degradation.

There are reports that provided microscopic evidence for the engulfment of entire plastids within the central vacuole [44,45,46], whereas other studies failed to confirm these observations (e.g., [47]). Although the available evidence suggests that engulfment of whole chloroplasts may be a relatively infrequent phenomenon, this does not rule out the involvement of the central vacuole. There is an early report on the late accumulation of amino acids (presumably arising from protein degradation) in the central vacuole of senescing leaf cells [48], and more recently Ono *et al*. [49] showed the accumulation of GFP in the central vacuoles of Arabidopsis plants expressing Rubisco-GFP. This implies that the central vacuole may participate in chloroplast protein degradation, either by accomplishing the complete degradation of proteins to their constituent amino acids, or part of this process. In any event, the operation of the entire or partial breakdown of chloroplast proteins in the central vacuole would require transport mechanisms between both organelles.

## 4. Senescence-Associated Vacuoles: Contents, Characteristics, Markers

In recent years evidence has accumulated showing the co-existence of different classes of vacuoles in the same cells. For example, primary and secondary vacuoles can be detected in aleurone layer cells [50], whereas functionally specialized CAM-associated and sodium storage vacuoles co-exist in mesophyll cells of Mesembryanthemum [51]. In general, the occurrence of two different vacuole types in the same cell, although clearly not universal, is possible under certain physiological or developmental transitions [52]. This is the case in senescing leaf cells, where in addition to the central vacuole, there are smaller vacuoles, absent from non-senescing leaves, and therefore termed “senescence-associated vacuoles” (SAVs) [53]. SAVs have been detected in soybean, Arabidopsis and tobacco [35,53,54]. The number of SAVs increases dramatically as senescence progresses, reaching relatively large numbers (40–60 SAVs per cell in a 1 µm thick optical section) during the period when chloroplast protein loss is most intense [35,55]. Further strengthening the association of SAVs with senescence, the number of SAVs per cell section increases when senescence is accelerated by ethylene, and, conversely, SAVs are almost non-detectable in leaves treated with cytokinins [35,54]. It is noteworthy that SAVs are detected in chloroplast-containing cells, *i*.*e*., mesophyll, and, to a lesser extent in guard cells, but they seem to be absent from the rest of the epidermis [53].

SAVs contain vacuolar H^+^ pyrophosphatase in their limiting membrane, are acidic, and display no organized internal structure [53], which justifies their assignment as a novel class of vacuoles. However, there are a number of features distinguishing SAVs from the central vacuole. As mentioned before, SAVs are smaller (0.5–0.8 µm in diameter). Furthermore, SAVs are more acidic than the central vacuole. Direct measurements with the pH sensor probe Lysosensor Yellow/Blue shows SAVs to have a lumenal pH around 5.2, whereas the pH of the central vacuole is approximately 6.0 [53]. SAVs membranes also lack the γ-TIP aquaporin, which is diagnostic of the central vacuole and may be important for internal equilibration of water [40].

**Figure 1:**
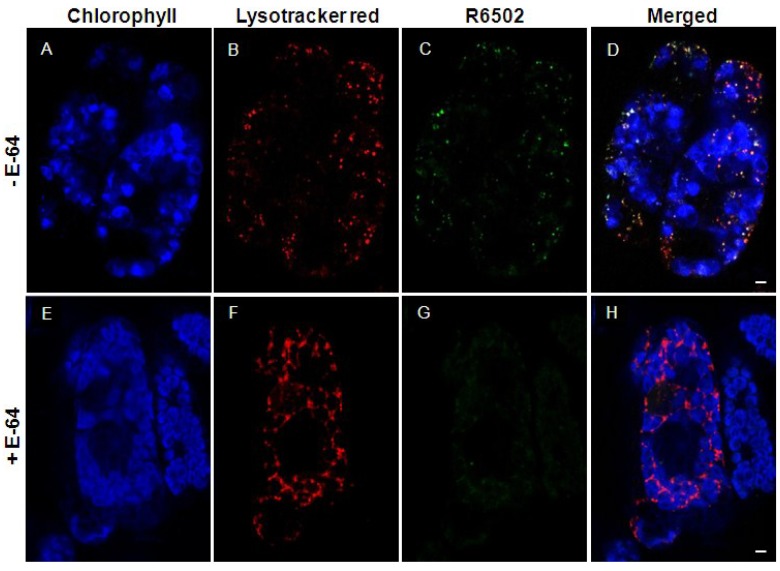
*In vivo* inhibition of cysteine protease activity of SAVs with the specific inhibitor E-64. Cells were isolated from senescing (3 days) tobacco leaves, and treated for 2 h with E-64 (100 µM, E through H), or left in buffer as controls (**A** through **D**). Cells were later stained with R6502, a probe for protease activity that becomes brightly fluorescent upon cleavage (**C** and **G**) and Lysotracker Red, an acidotropic marker for acid organelles (**B** and **F**). Panels A and E show chlorophyll autofluorescence, whereas panels D and H show a merged image of Chl, Lysotracker Red and R6502. Co-localization between Lysotracker Red and R6502 is pseudocolored white. Bars represent 5 µm. Redrawn from [54].

**Figure 2:**
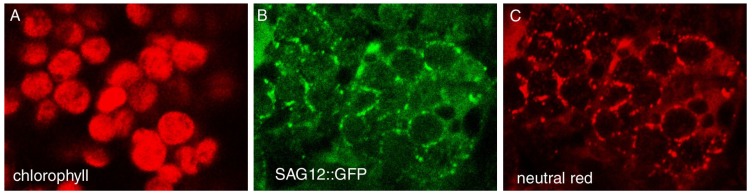
SAG12-GFP localization in senescing Arabidopsis leaves. Confocal images through the mesophyll of a senescing leaf from a SAG12-GFP transgenic plant incubated with the acidotropic dye Neutral Red. (**A**) Chlorophyll fluorescence (excitation 633 nm/emission > 650 nm), (**B**) SAG12-GFP (excitation 488 nm/ emission 505–550 nm), (**C**) Neutral Red (excitation 543 nm/emission 550–605 nm). Note that the GFP signal colocalizes with the fluorescence from Neutral Red, indicating that SAG12-GFP is located inside SAVs. Scale bar = 10 µm. Redrawn from [53].

A distinctive feature of SAVs is their high peptidase activity. When senescing leaf cells are incubated in the presence of a fluorescent probe (R6502) for peptidase activity, most of the fluorescence resulting from hydrolysis of the probe is detected in SAVs [35,53,54]. Since R6502 has been reported to detect cysteine type proteases with high efficiency [55] we surmised that SAVs peptidase activity was mostly due to cysteine-proteases [53], which was later confirmed by the observation that *in vivo* peptidase activity in SAVs is almost completely abolished by pre-incubation with the diagnostic cysteine protease inhibitor E-64 [54] (Figure 1). Likewise, subcellular fractionation combined with the use of an activity-based probe for cysteine proteases, DCG-04 [56,57] detected a large part of the cysteine protease activity of senescing cells in a fraction enriched in SAVs [54]. Up-regulation of cysteine protease genes is a common observation in many transcriptomic studies of leaf senescence in several plant species (reviewed in [14]), which suggests that SAVs contain some of the senescence up-regulated proteases of the cell. In line with this, the senescence-specific cysteine protease SAG12 appears to be located preferentially in SAVs [53,54] (Figure 2), confirming fluorescent (R6502) and activity-based probe (DCG04) results about the prevalence of cysteine proteases in these lytic vacuoles. The localization of a SAG12-GFP fusion in SAVs is quite significant in view of the strict senescence-associated expression of SAG12 [58,59,60], and this makes SAG12-GFP a convenient and specific marker for SAVs. Consistent with this, increased expression of SAG12 parallels the accumulation of SAVs during senescence of tobacco leaves [54]. SAG12 may account for part of the proteolytic activity of SAVs, but it is clearly not required for SAVs biogenesis [53].

Summing up, the available evidence suggests that at least two types of vacuoles co-exist in senescing mesophyll cells, the central vacuole, and a large number of smaller, senescence-associated vacuoles loaded with high peptidase activity.

## 5. Involvement of SAVs in Chloroplast Protein Degradation

**Figure 3:**
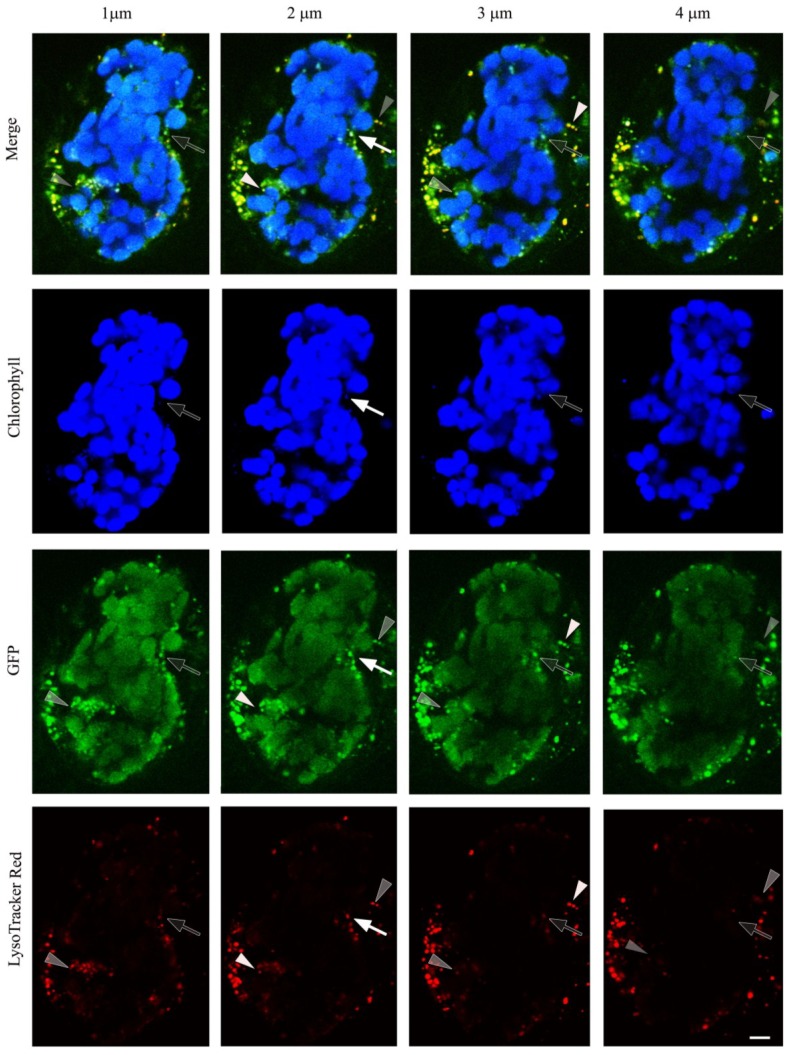
Z-stack (consecutive 1 µm thick optical slices) covering a depth of 4 µm in a mesophyll cell of a senescing tobacco leaf expressing GFP targeted to the plastid [61]. As an example, SAV detected in the middle planes (second or third optical slices) are indicated with white arrows (one SAV with LysoTracker Red and GFP and Chl fluorescence) or white arrowheads (two SAVs with LysoTracker Red, and GFP fluorescence). Empty arrows/arrowheads show fainter signals above or below the focal plane of selected SAVs. Note that most SAVs, stained by Lysotracker Red, also contain GFP, and that almost all GFP located outside chloroplasts is found in SAVs. Scale bar: 5 µm. Redrawn from [35].

Their high peptidase activity and their occurrence in chloroplast-containing cells (*i*.*e*., mesophyll and guard cells), led us to speculate that the breakdown of chloroplast proteins during senescence might partially occur in SAVs. Two lines of evidence point to the relocation of some chloroplast proteins to SAVs in senescing leaf cells. First, in a transgenic line of tobacco expressing GFP fused to the recA transit peptide for targeting to plastids, GFP fluorescence is restricted to the chloroplasts in mature leaves [35,61], while in senescing cells, in addition to the presence of GFP in chloroplasts, also SAVs (detected by their staining with the acidotropic marker Lysotracker Red) contain GFP (Figure 3) [35]. This suggests that some chloroplast proteins relocalize to SAVs in the course of senescence. A second evidence for the presence of chloroplast proteins in SAVs comes from subcellular fractionation and isolation of a SAVs-enriched fraction. The large subunit of Rubisco and glutamine synthetase II were readily detected in isolated SAVs confirming the relocation of chloroplast proteins to SAVs during senescence. The finding of the large subunit of Rubisco is significant, not only because this is the most abundant protein in C3 plants, but also because the large subunit is chloroplast-encoded and synthesized within the plastid, therefore the most likely explanation for its presence in SAVs is relocation. The lack of characteristic PSII proteins, such as D1 and Lhcb in this SAVs-enriched fraction indicates also that the presence of Rubisco is not due to contamination, and, perhaps more important, that PSII proteins are degraded by a different proteolytic pathway.

Since SAVs contain high peptidase activity, it is not surprising that isolated SAVs can degrade the chloroplast proteins (e.g., Rubisco) contained within them in autodigestion experiments [35,54]. About 25% of Rubisco contained in SAVs is lost in 4 h of incubation, and this moderate rate of degradation possibly explains why chloroplast-targeted GFP is relatively stable in SAVs, allowing for its detection through confocal microscopy [35,54]. The loss of Rubisco in autodigestion experiments is reduced to a large extent by addition of cysteine protease inhibitors, whereas serine protease inhibitors are largely ineffective [35,54]. Thus, *in vitro* proteolytic activity of SAVs seems to be due to cysteine proteases, which is consistent with all the observations indicating that cysteine proteases are an important component of SAVs. Correlative evidence suggests that Rubisco degradation by SAVs cysteine proteases also occurs *in vivo*. Incubation of wheat leaf segments [62] and tobacco leaf disks [54] with an inhibitor of cysteine proteases (E64) reduces the degradation of chloroplast proteins, including Rubisco. This correlates with severe inhibition of the cysteine protease activity of SAVs [54]. Although a direct genetic approach to test whether chloroplast proteins are degraded in SAVs is still lacking, the presence of chloroplast proteins in SAVs and the decreased protein degradation by treatments that block SAVs proteolytic activity lend support to the idea that SAVs may be part of a proteolytic pathway(s) involved in breakdown of the stromal proteins of chloroplasts in senescing leaves.

## 6. SAVs and Rubisco-Containing Bodies, Is There a Link?

Almost in parallel to the finding of SAVs, Rubisco-Containing Bodies (RCBs) were detected in senescing wheat leaves [33,34]. RCBs were first described as small (0.4–1.2 µm in diameter), double membrane bound vesicles detected in the cytosol of leaf cells through transmission electron microscopy [33]. Their numbers increased during senescence, and immunolocalization experiments showed that they contained chloroplast stromal proteins, such as Rubisco and glutamine synthetase II, but not thylakoid proteins [33]. This is similar to what we know about SAVs, although SAVs are apparently bound by a single membrane [53]. On the other hand, no evidences on the presence of proteases or peptidase activity within RCBs have been reported, which is an important difference with respect to SAVs. Testing for peptidase activity in RCBs would be important to confirm their role as a transport (not lytic) vesicle carrying stromal proteins to the central vacuole, and to differentiate RCBs functions from those of SAVS. Formation of RCBs depends on the operation of the autophagic pathway [34,46]. In transgenic lines expressing GFP or DsRed targeted to the plastid, RCBs are detected in the central vacuole of concanamycin-Atreated cells [34,46], possibly because concanamycin-A, an inhibitor of vacuolar H^+^ ATPase, raises vacuolar pH thereby reducing its hydrolytic activity.

**Figure 4:**
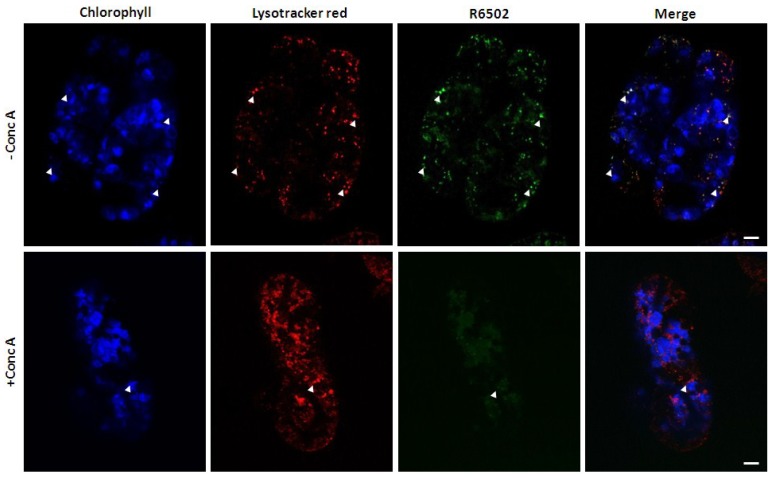
Inhibition of SAVs proteolytic activity by concanamycin-A. Detached tobacco leaves were induced to senesce by incubation in darkness for 3 days, then 5-mm diameter disks were excised and incubated in 1 µm concanamycin-A for 15 h. Cells were stained with Lysotracker Red and R6502 and observed through laser confocal microscopy. Note the almost complete disappearance of the characteristic punctuate pattern of R6502 (*i*.*e*., proteolytic activity) corresponding to SAVs in cells from disks treated with concanamycin-A (lower panels).

**Figure 5:**
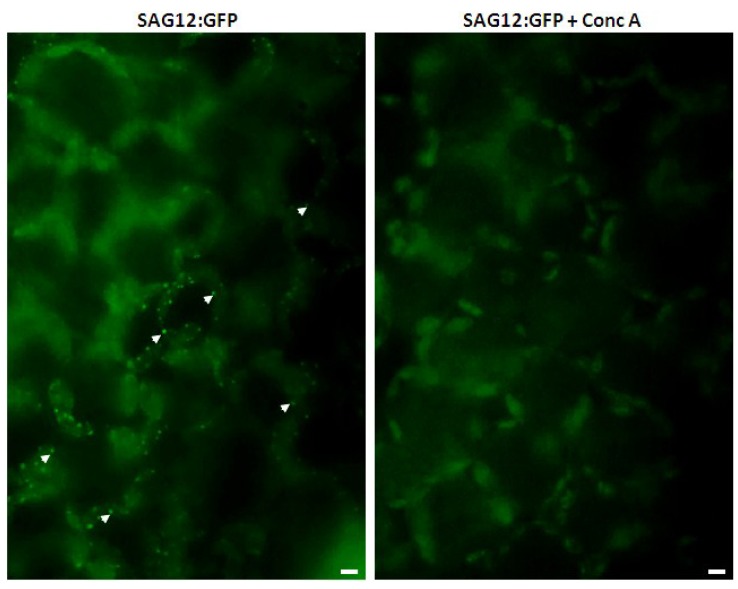
Disappearance of the SAG12-GFP signal due to concanamycin-A treatment. Detached tobacco leaves expressing SAG12 fused to GFP were induced to senesce by incubation in darkness for 3 days. Leaf disks were then excised and incubated in 1 µm concanamycin-A for 15 hours. Leaf pieces were observed directly with an epifluorescence microscope (Olympus BX61) fitted with an FITC filter. Note that the bright fluorescent vesicles (SAVs, a few representative SAVs are indicated by arrowheads) in the control, left panel, disappeared completely in leaf cells treated with concanamycin-A (right panel).

Since there seem to be a number of similarities between SAVs and RCBs, one may wonder whether they are two different and completely independent structures, or two different but somehow interrelated vesicles, or even whether they are the same structure given different names. Dependence of RCBs on the autophagic pathway and inhibition of SAVs by concanamycin-A treatment are two important differences which suggest these might be different structures representative of two different proteolytic pathways. We previously showed that small lytic vacuoles stained by the acidotropic dye Lysotracker Red and the cysteine protease probe R6502 accumulate normally in an *atg7* knock out mutant [53], indicating that formation of SAVs does not depend on the autophagic pathway. Regarding concanamycin-A, detection of RCBs is facilitated by pre-treatment of leaf disks for 20 h with concanamycin-A [34,46]. On the contrary, in our experiments with tobacco, concanamycin-A significantly reduced the staining of SAVs by R6502, *i*.*e*., their proteolytic activity became almost non-detectable [63] (Figure 4). This might be simply explained on the basis of the operation of vacuolar H^+^ ATPases in the acidification of SAVs lumen. Pre-treatment with concanamycin-A might render SAVs less acidic and, since many cysteine proteases have an acidic pH optimum, labeling by R6502 might be impaired by concanamycin-A. However, concanamycin-A treatment also decreases the number of SAVs detected through fluorescence of GFP in a line expressing a SAG12-GFP fusion [63] (Figure 5). The disappearance of SAG12-GFP is hard to attribute to increased SAVs pH, on the contrary, since GFP is reportedly less stable under low pH [64], an increase of the highly acidic pH of SAVs should increase GFP stability and, therefore, improve SAG12-GFP detection. Alternatively, inhibition of SAVs by concanamycin-A could be interpreted as evidence for the involvement of the Golgi apparatus in SAVs biogenesis, as concanamycin-A is known to disrupt trafficking through the trans-Golgi network [65], In any event, the scant evidence available so far, *i*.*e*., dependence on the autophagic pathway and response to concanamycin-A, suggests that RCBs and SAVs may be different entities.

If SAVs and RCBs turned out to be different structures involved in stromal protein degradation, then a relevant question would be whether they co-exist in senescing cells or if they represent different breakdown pathways operating in specific developmental scenarios. This is a difficult question without more information on their molecular characteristics to allow for their simultaneous identification in living cells. However, it is worth noting that in tobacco leaf cells expressing GFP-targeted to the chloroplast, almost all vesicular structures containing GFP outside plastids are labeled by Lysotracker Red, *i*.*e*., they are acidic (Figure 3), and in wild type cells almost all Lysotracker Red labeled SAVs contain high peptidase activity, which are distinctive features of SAVs. This might imply that, under certain conditions, SAVs may be quite prevalent. On the other hand, based on calculations of the amount of GFP released into the central vacuole in a line expressing Rubisco fused to GFP, Ono *et al*. [49] calculated that at least 41% of Rubisco might be degraded through an autophagic pathway involving RCBs. The independent operation of SAVs and RCBs is also suggested by the phenotype of autophagy knock out mutants, in which chloroplast protein degradation proceeds normally, or in many cases is accelerated, rather than delayed [66,67,68]. A careful quantitative analysis of Rubisco levels in senescing leaves of Arabidopsis shows that the time-course of Rubisco loss is very similar in wild type and two autophagy mutants, except for a slight retention of Rubisco very late during senescence (*i*.*e*., when leaves have already lost more than 95% of their chlorophyll [69]. Recent studies clearly show the accumulation of degradation fragments of Rubisco and glutamine synthetase II in an *atg5* knock out line [70]. Since RCBs presumably do not operate in an *atg5* KO, this suggests the operation of an alternative proteolytic pathway. SAG12 expression increases in some *atg* mutants [71], therefore SAVs might represent the pathway alternative to autophagy bringing about chloroplast protein degradation in autophagy mutants.

## 7. Future Directions

The available evidence indicates that Senescence-Associated Vacuoles are lytic compartments possibly involved in the degradation of chloroplast proteins during senescence. There are a number of questions that still need to be resolved, and these include, among others, (1) the biogenesis of SAVs, (2) the identification of SAVs proteases responsible for protein degradation, (3) an assessment of the quantitative importance of SAVs in chloroplast protein breakdown and (4) a detailed study of the interrelations between SAVs and other extraplastidial chloroplast degradation pathways. Undoubtedly, identifying molecular targets to impair SAVs biogenesis and/or function will be critical to facilitate progress in the understanding of this novel cellular structure.
